# Genomic Epidemiology of Shiga Toxin-Producing *Escherichia coli* Isolated from the Livestock-Food-Human Interface in South America

**DOI:** 10.3390/ani11071845

**Published:** 2021-06-22

**Authors:** Nicolás Galarce, Fernando Sánchez, Beatriz Escobar, Lisette Lapierre, Javiera Cornejo, Raúl Alegría-Morán, Víctor Neira, Víctor Martínez, Timothy Johnson, Danny Fuentes-Castillo, Elder Sano, Nilton Lincopan

**Affiliations:** 1 Departamento de Medicina Preventiva Animal, Facultad de Ciencias Veterinarias y Pecuarias, Universidad de Chile, Santiago 8820808, Chile; fernando.sanchez@ug.uchile.cl (F.S.); beatrizescob@gmail.com (B.E.); llapierre@uchile.cl (L.L.); jacornej@uchile.cl (J.C.); ralegria@veterinaria.uchile.cl (R.A.-M.); victorneira@u.uchile.cl (V.N.); 2 Programa de Doctorado en Ciencias Silvoagropecuarias y Veterinarias, Facultad de Ciencias Veterinarias y Pecuarias, Universidad de Chile, Santiago 8820808, Chile; 3 Facultad de Ciencias Agropecuarias y Ambientales, Universidad Pedro de Valdivia, Santiago 8370007, Chile; 4 Departamento de Fomento de la Producción Animal, Facultad de Ciencias Veterinarias y Pecuarias, Universidad de Chile, Santiago 8820808, Chile; vmartine@uchile.cl; 5 Department of Veterinary and Biomedical Sciences, College of Veterinary Medicine, University of Minnesota, St. Paul, MN 55108, USA; tjj@umn.edu; 6 Departamento de Patología, Faculdade de Medicina Veterinária e Zootecnia, Universidade de São Paulo, São Paulo 05508-270, Brazil; dannyfuentesmv@gmail.com; 7 Departamento de Microbiología, Instituto de Ciências Biomédicas, Universidade de São Paulo, São Paulo 05508-900, Brazil; eldersano@icb.usp.br (E.S.); lincopan@usp.br (N.L.)

**Keywords:** STEC, cattle, beef, molecular epidemiology, whole-genome sequencing, MLST, South America, One Health

## Abstract

**Simple Summary:**

Shiga toxin-producing *Escherichia coli* (STEC) are zoonotic pathogens that cause food-borne diseases in humans, where cattle and derived products play a key role as reservoirs and vehicles. We analyzed the genomic data of STEC strains circulating at the livestock-food-human interface in South America, extracting clinically and epidemiologically relevant information (serotypes, virulome, resistance genes, sequence types, and phylogenomics). This study included 130 STEC genomes obtained from cattle (*n* = 51), beef (*n* = 48), and human (*n* = 31) samples. The successful expansion of O157:H7 (ST11) and non-O157 (ST16, ST21, ST223, ST443, ST677, ST679, ST2388) clones is highlighted, suggesting common activities, such as multilateral trade and travel. Circulating STEC strains analyzed exhibit high genomic diversity and harbor several genetic determinants associated with severe illness in humans, highlighting the need to establish official surveillance of this pathogen that should be focused on detecting molecular determinants of virulence and clonal relatedness, in the whole beef production chain.

**Abstract:**

Shiga toxin-producing *Escherichia coli* (STEC) are zoonotic pathogens responsible for causing food-borne diseases in humans. While South America has the highest incidence of human STEC infections, information about the genomic characteristics of the circulating strains is scarce. The aim of this study was to analyze genomic data of STEC strains isolated in South America from cattle, beef, and humans; predicting the antibiotic resistome, serotypes, sequence types (STs), clonal complexes (CCs) and phylogenomic backgrounds. A total of 130 whole genome sequences of STEC strains were analyzed, where 39.2% were isolated from cattle, 36.9% from beef, and 23.8% from humans. The ST11 was the most predicted (20.8%) and included O-:H7 (10.8%) and O157:H7 (10%) serotypes. The successful expansion of non-O157 clones such as ST16/CC29-O111:H8 and ST21/CC29-O26:H11 is highlighted, suggesting multilateral trade and travel. Virulome analyses showed that the predominant *stx* subtype was *stx2a* (54.6%); most strains carried *ehaA* (96.2%), *iha* (91.5%) and *lpfA* (77.7%) genes. We present genomic data that can be used to support the surveillance of STEC strains circulating at the livestock-food-human interface in South America, in order to control the spread of critical clones “from farm to table”.

## 1. Introduction

Shiga toxin-producing *Escherichia coli* (STEC) is a zoonotic diarrheagenic pathotype of *E. coli* with the common characteristic of producing cytotoxins of the Shiga toxin family (Stx) [1]. Although their participation in different diarrheal processes in animals is recognized, their frequency is low; therefore, they are considered as reservoirs and disseminators of STEC. Thus, gut colonization favors transmission to other animals, the environment, and humans [2].

Cattle, which lack cellular receptors for Stx [3], are asymptomatic carriers of STEC strains and are recognized as the main source for human STEC infections. Most outbreaks are associated with the consumption of undercooked beef or unpasteurized dairy products [4]. In addition, some animals known as “super-shedders” can excrete more than 10^4^ CFU/g feces [5], increasing the probability of infection in other animals and contamination of meat. Reported prevalence of STEC in cattle in the last 10 years in South America has ranged from 14% to 90% [6,7,8,9]. Given that the presence of this pathogen is frequent in cattle feces, their carcasses may become contaminated during slaughtering operations, leading to meat products containing STEC. Thus, one of the most common STEC vehicles associated with outbreaks is beef [10,11]. Therefore, preventing the introduction of this pathogen into the food chain represents a serious challenge for food safety and health official agencies [12]. In this context, in the USA serogroups O26, O45, O103, O111, O121, and O145 (also known as “the big six”), alongside O157 have been considered adulterants in raw, non-intact ground beef products since 2011 [13]. Nevertheless, the presence of STEC in soil, water, and animal manure are factors associated with the contamination of other foods such as vegetables [14,15].

In humans, STEC infections can cause severe gastroenteritis, hemorrhagic colitis (HC), and life-threatening hemolytic-uremic syndrome (HUS), mainly in children under five years of age [16]. Foodborne STEC infections cause more than one million cases of illness worldwide per year, with approximately 3890 HUS cases and 230 deaths [17,18]. In South America, STEC infections remain endemic and have contributed to the burden of acute diarrheal syndrome in this region, with a significant number of HUS cases mainly in Argentina, Chile, Uruguay, and Brazil [19,20,21]. This could be explained by the high beef consumption in those countries [22]; the cattle biomass in South America, which in 2018 was 357,222,668 animals, mainly concentrated in Brazil (59.8%), Argentina (15.1%), and Colombia (7.2%) [23]; and by the presence of hypervirulent clones circulating in the cattle-beef-human interface [24]. In this sense, a previous study in Argentina characterized a collection of STEC O157 strains isolated from cattle and humans; reporting that 98% of the strains harbored molecular determinants associated with severe illness in humans [25], a situation not reported anywhere before. Nevertheless, there are no other studies addressing the phylogenomics of STEC strains in South America; a situation that highlights the need to conduct robust analysis that can characterize and geographically track STEC strains circulating in the animal-food-human interface, as a strategy to promote animal and public health, under the concept of One Health.

Although epidemiological studies on STEC distribution have shown that O157:H7 serotype has been most frequently associated with outbreaks [26], “big-six” non-O157 STEC serogroups have also been associated with severe illness in humans worldwide [5]. However, most STEC strains isolated from cattle in South American countries do not belong to the aforementioned serogroups [8,27,28,29,30]. Given these contradictory reports, the isolation and serotype rates of STEC strains isolated from cattle are insufficient to explain the high HUS incidence in South American countries [19]. Moreover, a high genotypic diversity has been reported within serotypes [12], and horizontal gene transfer of the O-antigen can occur among different *E. coli* strains [31], making the prediction of the virulence of a strain based on its serotype inaccurate [32]. Therefore, additional analyses are required, such as virulome, resistome, and MLST patterns of the circulating strains.

The primary virulence factor of STEC is Stx, which is classified into two types, Stx1 and Stx2. Each type is further classified into several subtypes (*stx1a*, *stx1c*-*e*, *stx2a*-*l*) [33]. The typing and subtyping of Stx is necessary to predict the virulence of the strain, as Stx2 has been shown to be more virulent than Stx1, and is more frequently associated with HC and HUS, as well as the Stx2a, Stx2c, or Stx2d subtypes [5]. Additionally, STEC may harbor other virulence factors that contribute to the development of severe illness in humans, such as the *eae* gene, which is located in the locus of enterocyte effacement (LEE) [5]; or the *saa* gene [34] and the locus of adhesion and autoaggregation (LAA) [35] in the LEE-negative strains. More recently, convergence of virulence and antimicrobial resistance (AMR) among STEC strains has emerged as a worrying problem that threatens animal and public health globally. Indeed, STEC strains exhibiting a multidrug-resistant profile have been identified worldwide, including South America [36,37,38,39,40,41]. International trade of food and animals as well as international travelers can lead to the introduction of highly virulent and antimicrobial resistant strains [12,42,43,44], modifying the traditional epidemiology of the pathogen, and challenging biosecurity and surveillance protocols.

Given the high variety of serogroups, virulotypes and resistotypes, and the intrinsic genomic flexibility of *E. coli*, whole genome sequencing (WGS) is a specific and sensitive method to conduct epidemiological and phylogeographic studies. Several genomic studies have been carried out to determine the virulome, resistome, and the worldwide spread of STEC clones isolated from animals, the environment, food, and humans [32,45,46,47]. In South American countries these studies are scarce, and have been mainly focused on virulence determinants associated with HC and HUS [35,48].

Genomic surveillance of STEC strains is necessary to improve the knowledge about the dynamic of dissemination, and of the role of cattle as reservoirs and disseminators of STEC lineages associated with severe disease in humans. Thus, the aim of this study was to analyze genomic data of STEC strains isolated from cattle in Chile, and to perform a comparative genomic analysis of STEC strains circulating at the cattle-beef-human interface in South America, extracting clinically and epidemiologically relevant information, including virulome, antimicrobial resistance genes, sequence types, clonal complexes, and phylogenomics, in order to provide relevant epidemiological data to surveillance programs at national and international level, under a One Health approach.

## 2. Materials and Methods

### 2.1. Bacterial Isolates and Whole-Genome Sequencing (WGS)

Twenty-one STEC strains isolated from cattle were sequenced in this study, of which twenty were isolated in 2018 from cattle at abattoirs in the Región Metropolitana of Chile [8], and one was isolated in 2019 from cattle raised in a backyard production system in the same region [49]. Genomic DNA of these 21 STEC strains was extracted using the Wizard Genomic DNA purification kit (Promega, Madison, WI, USA), following the manufacturer’s instructions. Genomic DNA libraries were constructed using the QIAseq FX DNA library kit (Qiagen, Hilden, Germany). Sequencing was performed using the MiSeq Reagent kit v3 600 cycles on an Illumina MiSeq platform (Illumina, San Diego, CA, USA). All raw FASTQ files were uploaded and processed through the QAssembly (v3.61) automated assembly pipeline on the Enterobase platform (http://enterobase.warwick.ac.uk). De novo assemblies that passed quality control with the standard established by Enterobase for *E. coli*/*Shigella* were used [50]. Contigs shorter than 200 nt were removed and sequences were deposited at GenBank under BioProject numbers PRJNA656305 and PRJNA682583.

### 2.2. Publicly Available Genome Sequences

For comparative analysis, all *E. coli* strains isolated in any South American country from cattle, beef, and people, whose FASTQ files were available at SRA GenBank (https://www.ncbi.nlm.nih.gov/genbank/) were downloaded on 6 November 2020. These 600 whole genome sequences were mapped using BWA [51] on the sequences of every *stx* type/subtype. Thus, a total of 109 genomes of *stx*-positive *E. coli* strains were selected for comparative analysis with the 21 STEC genomes obtained in this study (Table S1).

### 2.3. Virulome and AMR Gene Analysis

The prediction of virulence genes was performed on VirulenceFinder 2.0 (https://cge.cbs.dtu.dk/services/VirulenceFinder/) and ABRicate (v.0.8.13) (https://github.com/tseemann/abricate/). The assessed virulence genes included *aggR* (aggregative adherence transcriptional regulator), *cdtB* (cytolethal distending toxin), *eae* (intimin), *efa1*(adhesion and invasion of epithelial cells), *ehaA* (adhesion to epithelial cells), *ehxA* (enterohemolysin), *hlyA* (α-hemolysin), *hra* (intimate attachment to microvilli), *iha* (adhesion and invasion of epithelial cells), *lpfA* (adhesion to fibronectin, laminin, and collagen), *subA* (subtilase cytotoxin), *tia* (adhesion and invasion of epithelial cells), and *stx* subtypes. In the case of *saa* and *sab* (both related to adhesion to epithelial cells) genes, as well as LAA and some of its associated genes, including *ag43* (adhesion, autoaggregation, and biofilm formation), *hes* (adherence to epithelial cells), *lesP* (serine protease), and *pagC*-like (putative serum resistance), were screened through BLASTn (https://blast.ncbi.nlm.nih.gov/Blast.cgi), according to protocols described in a previous study [48]. ResFinder 4.1 (https://cge.cbs.dtu.dk/services/ResFinder/) was used to identify the AMR genes. These analyses were performed with a default setting of 90% of identity threshold and 60% minimum gene length overlap. The presence of these genes was confirmed when a coverage and identity >90% was achieved.

### 2.4. Epidemiological Typing and Phylogenomic Analysis

The multilocus sequence typing (MLST) scheme of Achtman was used to identify sequence types (STs) and clonal complexes (CCs) on the Enterobase platform (http://enterobase.warwick.ac.uk). We have additionally performed a systematic search of scientific literature containing MLST data of STEC strains identified in South American countries. From genome and literature data, we have constructed a map containing the distribution of STs of STEC strains circulating at the livestock-beef-environment-human interface in South America [21,39,52,53,54,55,56]. The SerotypeFinder 2.0 tool (https://cge.cbs.dtu.dk/services/SerotypeFinder/) was used to determine serotype [57]. A minimum spanning tree was constructed in Enterobase with the MSTree V2 algorithm and the wgMLST scheme (http://enterobase.warwick.ac.uk) for phylogenomic analysis of all STEC genomes. The phylogenetic tree was generated with interactive Tree of Life, iTOL v6 (https://itol.embl.de). CSI Phylogeny 1.4 (https://cge.cbs.dtu.dk/services/CSIPhylogeny) was used with default settings to generate an approximately maximum-likelihood phylogenetic tree with the 21 genome assemblies obtained from this study, using *E. coli* strain K-12 MG1655 (RefSeq accession number NC_000913.3) as reference genome. The percentage of reference genome covered by all isolates was 81.24%, corresponding to 3,771,065 positions found in all genomes. iTOL v6 (https://itol.embl.de) was used to root the tree at midpoint and annotate the tree with data from Enterobase and genomic data.

### 2.5. Statistical Analysis

A logistic multivariable regression model was performed to assess the association between *stx* type (dependent variable) and the geographic location and isolation source of the strains included in this study (independent variables), where Y (response) can have only two values (Y = 0 or Y = 1) [58]. Three models were developed, one for each *stx* type gene and one for the presence of both *stx* types at the same time. To ensure adequate convergence of the model and increase the statistical power of the results, epsilon was set at a high level (epsilon = 1 × 10^−6^). The fit of the models to the data was evaluated with the Hosmer–Lemeshow test [59]. The variable geographic location was modified to fit into the analysis by collapsing the isolates from Uruguay, Paraguay, and Ecuador into a new variable defined as other. All the statistical analyses were performed using R 4.0.2 (The R Foundation, Vienna, Austria) [60].

## 3. Results

### 3.1. Source and Origin of the Analyzed STEC Genomes

In total 130 STEC genomes were analyzed, of which 21 were obtained in this study. Genomes were representative of STEC isolates recovered from cattle (39.2%), beef (36.9%), and humans (23.8%). Analyzed STEC genomes were from Chile (*n* = 88), Argentina (*n* = 30), Ecuador (*n* = 7), Paraguay (*n* = 3), and Uruguay (*n* = 2). Table S2 contains the source, origin, and isolation date of each analyzed STEC strain.

### 3.2. Virulome and Antibiotic Resistome of STEC Strains

All strains met the WGS quality parameters of Enterobase, with a total average of filtered reads of 419,230.8 and an average coverage of 28.6 (Table S1). Among the 130 STEC genomes, 72.3% (*n* = 94) harbored the *stx2* gene alone, 20.8% (*n* = 27) were *stx1*+*stx2* positive, and 6.9% (*n* = 9) harbored *stx1* alone. The *stx* subtype most frequently detected was *stx2a* (54.6%, *n* = 71), followed by *stx1a* (26.9%, *n* = 35), *stx2c* (22.3%, *n* = 29), *stx2d* (16.2%, *n* = 21), *stx2b* and *stx2g* (1.5%, *n* = 2), and *stx1c* (0.8%, *n* = 1). Among the other virulence genes assessed, the most frequently detected was *ehaA* (96.2%, *n* = 125), followed by *iha* (91.5%, *n* = 119), *lpfA* (77.7%, *n* = 101), *ehxA* (60%, *n* = 78), *hlyA* (58.5%, *n* = 76), *tia* (41.5%, *n* = 54), *saa* (40.8%, *n* = 53), *subA* (32.3%, *n* = 42), *eae* (30.8%, *n* = 40), *cdtB* (20%, *n* = 26), and *sab* (17.7%, *n* = 23). Additionally, 43.1% (*n* = 56) harbored LAA, with its associated genes *pagC*-like and *hes* (83.9%, *n* = 47), *ag43* (64.3%, *n* = 36), and *lesP* (28.6%, *n* = 16).

Studies focusing on the AMR of STEC strains isolated from the livestock-beef-human interface in South America are scarce [38,41,61,62,63]. In this study, genomic analysis revealed a low prevalence of resistance genes. In this regard, some strains carried aminoglycoside phosphotransferases encoding genes *aph(3″)-Ib* (3.1%, *n* = 4), *aph(6)-Id* (2.3%, *n* = 3), and *aph(3′)-Ia* (0.8%, *n* = 1), which reduce the electrostatic interaction of the drug and the bacterial rRNA [64]. Four strains harbored *tetA* or *tetB* genes, which encodes for tetracycline efflux proteins [65]. Three strains harbored *bla*_TEM-1_ β-lactamase encoding genes, which hydrolyze penicillins and narrow-spectrum cephalosporins [66]. Two strains harbored the *sul2* gene, which encodes a drug-resistant dihydropteroate synthase, reducing affinity with sulfonamides [67]. Two strains harbored the *fosA7* gene, encoding a fosfomycin inactivating enzyme [68], and two strains harbored the *qnrB19* gene, which encodes the Qnr protein that interacts with GyrA/B, preventing the action of fluoroquinolones [69]. As expected, all strains harbored chromosomal *bla*_AmpC_ [70] (Table S2).

### 3.3. Sequence Types, Serotypes and Phylogenomic Analysis of STEC Strains Circulating in South America

**Figure 1:**
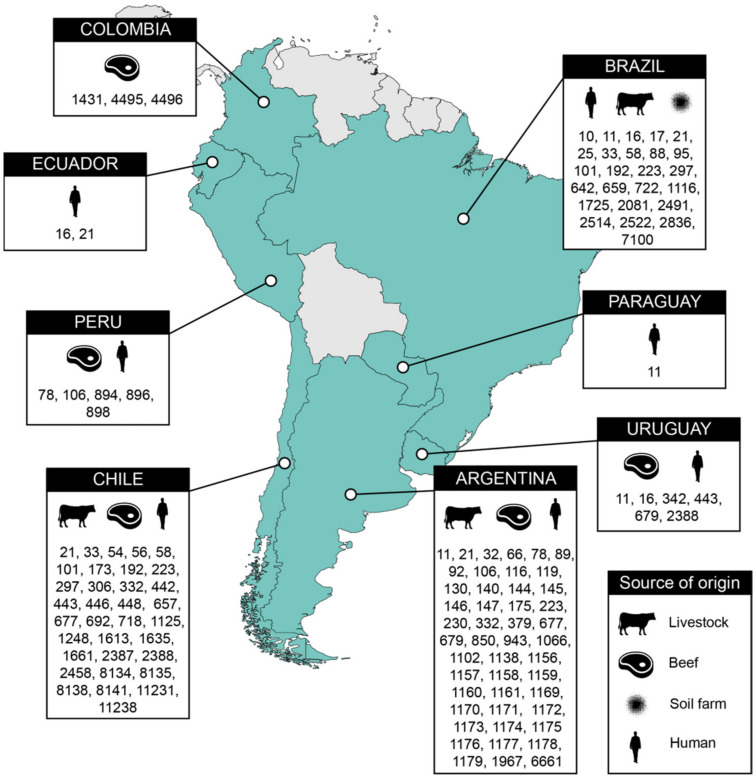
Distribution of sequence types (STs) of Shiga toxin-producing *Escherichia coli* (STEC) strains circulating at the livestock-beef-environment-human interface, in South America. In Brazil, for livestock origin, ST25, ST642, ST1725, ST2522, and ST7100 were isolated from sheep feces, whereas ST223 was isolated from bovine, and ST2491 was isolated from pig feces [21,39,52,53,54,55].

MLST typing revealed the presence of 41 STs among the circulating STEC strains, most of them belonging to ST11 (CC11) (20.8%, *n* = 27), followed by ST297 (9.2%, *n* = 12), and ST58 (CC155) (5.4%, *n* = 7). Figure 1 shows the distribution of the STs of STEC strains circulating in South America, based on analysis of publicly available genomes and data obtained from previous surveillance studies [21,39,52,53,54,55,56,61]. Of clinical interest, ST677, ST58, ST16, ST342, and ST11 carried multiple resistance genes. In addition to *bla*_AmpC_, STEC strains of ST677 and ST58 isolated from beef samples in Chile carried *aph(3″)-Ib*, *aph(6)-Id* and *tetB* (*n* = 2), *aph(3″)-Ib*, *aph(6)-Id*, *sul2* and *tetB* (*n* = 1), or *qnrB19* (*n* = 2) resistance genes; whereas human STEC strains belonging to ST16 and ST342, from Uruguay, carried *aph(3′)-Ia*, *aph(3″)-Ib*, *bla*_AmpC_, *bla*_TEM-1B_, *sul2* and *tetA* (*n* = 1), or *bla*_AmpC_ and *fosA7* (*n* = 2) resistance genes, respectively. Two human STEC belonging to ST11, from Paraguay, carried *bla*_AmpC_ and *bla*_TEM-1B_ genes.

Among the 130 STEC genomes analyzed, the most frequently detected serotypes were O-:H7 (10.8%, *n* = 14) and O157:H7 (10%, *n* = 13), followed by O113:H21 (9.2%, *n* = 12). Table S2 summarizes the STs, CCs, and serotypes from all STEC genomes analyzed.

**Figure 2:**
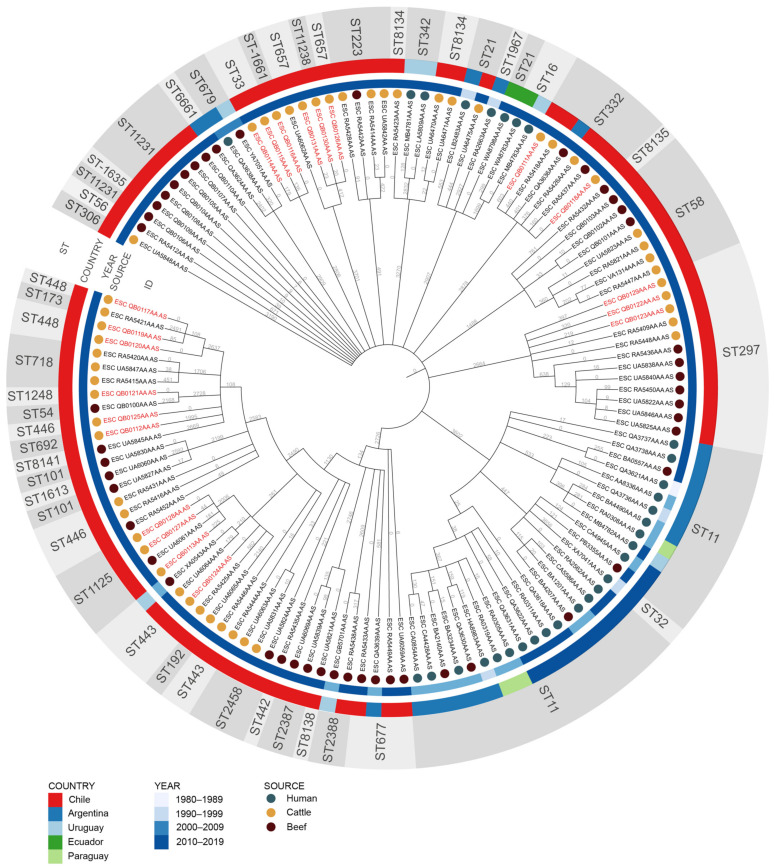
Minimum spanning tree for 130 STEC strains isolated from cattle, beef, and humans in South America, built by the MSTree V2 tool and wgMLST scheme from Enterobase. *Escherichia coli* sequence types (ST), country, year, and source of origin are compared. Genome IDs in red letters correspond to strains sequenced in this study. Numbers in the branches show the differences of wgMLST alleles between strains. An interactive version of the tree can be found at https://itol.embl.de/tree/179113207189378131606509422.

We further investigated the genomic relatedness among STEC lineages identified in this study with other STEC strains isolated in South America from human, cattle, and beef samples. The minimum spanning tree of the wgMLST analysis revealed that the 21 STEC strains sequenced in this study (genome IDs in red letters in Figure 2), clustered with other STEC strains previously isolated from cattle or beef in Chile, supporting the persistence of some clones circulating within the country, such as ST332, ST297, ST443, and ST1125 (Figure 2). On the other hand, some clusters included STEC of ST679 from beef (Uruguay) and human (Argentina), ST223 from beef and cattle (Chile), ST21 from human (Argentina) and cattle (Chile), ST332 from cattle (Chile) and beef (Chile and Argentina), and ST443 from beef (Uruguay) and cattle (Chile). For ST11, two epidemiologically relevant clusters were identified. The first included human strains from Argentina, Uruguay, and Paraguay, and the second cluster included STEC strains from human samples collected in Argentina and Paraguay and beef samples collected in Argentina (Figure 2).

**Figure 3:**
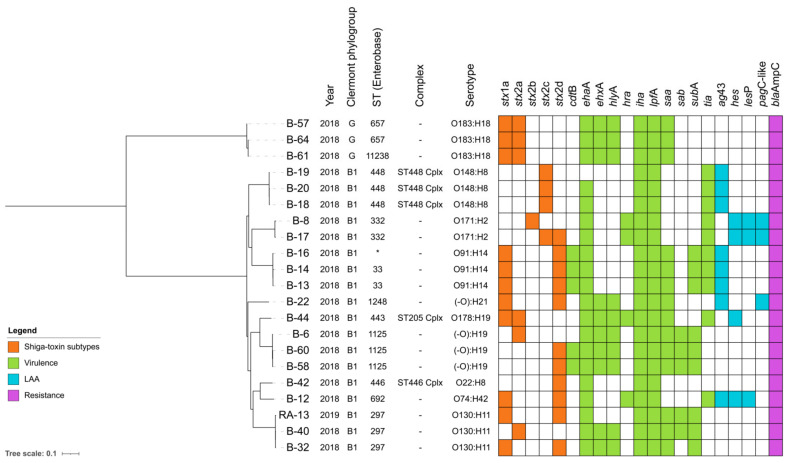
SNP-based phylogenetic tree of the 21 STEC strains isolated from cattle samples collected in Chile (this study). The heatmap was constructed based on Clermont phylogroup, multilocus sequence typing (MLST), serotype, and presence/absence of Shiga-toxin subtypes and virulence, LAA-associated genes, and resistance genes. * Strain B-16 belongs to the novel ST-1661, a single locus variant (SLV) of ST33 at the *fumC* allele. ST11238 is an SLV of ST657 at the *recA* allele. SNP matrix is quoted in Table S3.

SNP-based phylogenetic analysis confirmed genomic relatedness between STEC strains belonging to ST448/O148:H8 (B-18, B-19, and B-20, difference ≤ 246 SNPs), ST297/O130:H11 (B-32, B-40, and RA-13, difference ≤ 339 SNPs, ST1125/-O:H19 (B-6, B-58, and B-60, difference ≤ 238 SNPs), ST657/O183:h18 (B-61) and ST11238/O183:H18 (B-64) with difference of 124 SNPs, and ST33/O91:H14 and novel ST(-1661)/O91:H14 (B-13, B-15, and B-16, difference ≤ 431 SNPs). Additionally, most clonally related STs shared an identical virulome and resistome (Figure 3 and Table S3).

### 3.4. Association Between stx Type and the Geographic Location and Isolation Source of STEC Strains

The logistic regression model for *stx1* positivity showed a significant association with the isolation source; *stx*2 showed no significant association with any of the included variables, while for both *stx*1 and *stx*2 positivity showed a significant association with the geographic location of the sample (Table 1).

## 4. Discussion

Internationally, several studies have revealed a broad diversity of genomic patterns among STEC strains [32,47,71,72,73]. As far as we know, the present study is the first to analyze genomic patterns of STEC strains circulating in South America. Strains of the O157:H7 serotype have been shown to be genotypically diverse [74], with nine different clades identified by phylogenetic analysis [75]. Clades 6 and 8 are more frequently associated with severe disease in humans and with host-associated fitness [74,76,77]. However, this distribution may vary according to their geographic origin [78,79,80], suggesting a divergent evolution. On the other hand, non-O157 STEC strains show a broader phylogenomic diversity with significant differences in virulotypes [12,81,82]. Phylogenomic analysis corroborated these findings, where all O157 strains clustered together in the same clade, whereas non-O157 strains showed high diversity. All O-:H7 strains clustered together with the O157:H7 strains. This may have been caused by the horizontal transfer and replacement of a part or all of the O-antigen biosynthesis gene cluster from an original O157:H7 strain [31,83].

Serotypes O157:H7 and O113:H21 have been the most frequently detected in cattle, beef, other foodstuffs, and humans in South American countries [19,55,61,84,85,86]. In silico analysis confirmed that 96.3% of the O157:H7 strains were isolated in Argentina from beef and human clinical samples, while all O113:H21 strains were isolated in Chile from beef and cattle samples, suggesting selection and predominance of specific serotypes within each country.

ST11 (CC11), ST58 (155), and ST297 were the most frequently detected. Those STs were isolated worldwide from human and non-human hosts, and are associated with illness both in humans and animals [47,87]. While ST11 is the main clone identified from human infections in Argentina, Brazil, Uruguay, and Paraguay; lineages of CC29 (ST16 and ST21) have emerged in human infections in Argentina, Ecuador, Brazil, Uruguay, and Chile; the latter being further identified in cattle [21,39,52,53,54,55]. Further epidemiologically important data observed from MLST analysis was the successful expansion of the ST11/O157:H7 clone through Brazil, Uruguay, Paraguay, and Argentina, where it has caused severe human infections, including HUS [21]. In the same way, non-O157 STEC lineages of the CC29 (ST16 (O111:H8) and ST21 (O26:H11)) have been isolated from human infections in Argentina, Brazil, Uruguay, and Ecuador; where in Chile this clone was identified in cattle. Specifically, ST21 (O26:H11) strains have been recognized as an emerging HUS-associated STEC lineage in European and North American countries [88,89], where ST16 has been isolated from cases reporting recent travel abroad in England [47,89].

Some specific STs have been identified in two or three countries suggesting common activities, including multilateral trade and travel. The international STEC clone ST223/CC155 (O113:H21) was identified in cattle and beef in Chile (this study) and it has been previously identified in cattle, beef, and hamburgers in Argentina [55], and in cattle in Brazil [53]. In fact, ST223 has been isolated from environment, food, and clinical infections from various European countries as well [53,90]. Worryingly, in Argentina and Thailand, this clone has been associated with HUS [53].

STEC strains of the ST58 have been present in Chile (cattle) and Brazil (humans) [21,61], and have been isolated from cattle in Sweden [90]. Strikingly, as well as the ST223 (alleles *adk*-6, *fumC*-4, *gyrB*-4, *icd*-18, *mdh*-24, *purA*-8, *recA*-14), the ST58 (alleles *adk*-6, *fumC*-4, *gyrB*-4, *icd*-16, *mdh*-24, *purA*-8, *recA*-14) is a single-locus variant of the ST155 (alleles *adk*-6, *fumC*-4, *gyrB*-14, *icd*-16, *mdh*-24, *purA*-8, *recA*-14), which has been designated as the primary founder of the CC155. The ST prediction performed in this study identified the presence of the ST677 (O174:H21) clone in beef samples from Chile and Argentina, whereas ST443 (O178:H19) and ST2388 (O15:H27) were identified in cattle and beef samples from Chile and Uruguay. While ST679 (O163:H19) was predicted in beef and human STEC genomes from Uruguay and Argentina, respectively, previous studies have reported the presence of ST78 and ST106 in Peruvian children and Argentinean cattle [52,55]. In this regard, ST78, a common ST in clinical strains, has been a predominant STEC genotype among cattle and environmental strains [91]. ST101 and S297 were also predicted from genomes of STEC strains isolated from humans and beef in Brazil and Chile, respectively. On the other hand, while ST101 has been identified in food samples in South Korea [92], the ST297 has been a predominant lineage in cattle from Sweden [90], and in pigs, cattle, milk, and water from dairy farms in China [93].

Interestingly, the phylogenetic analysis performed to evaluate the genomic relatedness among STEC strains confirmed the presence of clusters that include clones circulating at the livestock-food-human interface, supporting the persistence, adaptation, and successful expansion of specific lineages across and within different countries.

The most detected *stx* type was *stx2*, which has been shown to be associated with more virulent STEC lineages and HUS. In fact, Stx2 presents an LD_50_ 400 times lower than Stx1 in murine models [94]. Among the Stx subtypes, Stx2a, Stx2b, Stx2c, or Stx2d, have been most frequently associated with the development of HUS in infected patients. Other Stx subtypes have been associated with mild illness in humans without complications or as asymptomatic carriers [3,95]. Additionally, Stx2a is epidemiologically associated with increased excretion levels of STEC O157 from cattle [96] and increased transmission between animals, presumably because it is more rapidly produced than other Stx subtypes and restricts cellular proliferation of bovine epithelial cells [97].

Another important virulence factor of STEC is the *eae* gene, which has traditionally been recognized as a virulence-marker, but here only 30.8% of strains harbored this gene. These *eae*-positive strains included ST11 lineages, the “big six” (O26:H11, O103:H2, O111:H8, O145:H25, and O145:H28) and other serotypes (O69:H11, O98:H21, O172:H25) considered emergent [98]. However, the burden of illness caused by LEE-negative STEC strains has recently increased in several countries, such as Argentina, Chile, and Paraguay [99]. In this study, most of the analyzed strains were LEE-negative, but harbored different adhesin-encoding genes, including *lpfA*, *ehaA*, and *saa*. LpfA correspond to a major fimbrial subunit protein that is able to interact with fibronectin, laminin, and collagen IV [100]; EhaA is an autotransporter protein related with biofilm and cellular aggregation; while Saa is considered an LEE-negative marker [48] increasing adhesion to HEp-2 cells nearly 10-fold [101]. Moreover, some recently acquired pathogenicity islands (PAI) could contribute to its adhesion, such as LAA. Those PAI can be present as a complete structure with their four modules: module I (*hes* and other genes), module II (*iha*, *lesP*, and others), module III (*pagC*-like and other genes), and module IV (*ag43* and other genes), or as an incomplete structure with some missing modules. Among analyzed strains, this PAI was identified in 43.1% of the strains, mostly as a complete structure. The acquisition of this PAI is probably a recent evolutionary event in STEC [35], which could have contributed to the emergence of highly virulent LEE-negative strains associated with HC and HUS, which are widely distributed in South American countries [25,52,102,103,104].

Other toxin-encoding genes detected were *ehxA*, *hlyA*, *subA*, and *cdtB*, these last two only being detected in LEE-negative strains. The α-hemolysin (HlyA) and the plasmid-encoded enterohemolysin (EhxA) are widely distributed in STEC strains and have been frequently associated with mild to severe illness in humans [105,106]. CDT causes irreversible G2/M arrest, inhibition of proliferation, and death of human endothelial cells and is frequently detected in LEE-negative strains associated with HUS [107]. SubAB is highly toxic for a range of cell types and induces vacuolization and temporarily protein synthesis inhibition [108], and has a synergic effect with Stx2 in human glomerular endothelial cells damage, contributing to the development of HUS [109]. Given these results, the STEC strains circulating in South America, despite their geographical origin and isolation source, pose a public health risk.

Although antibiotic treatment of STEC infections in humans is not recommended, as they may worsen the disease by inducing toxin-related tissue damage and symptoms in patients [1], STEC can easily exchange AMR encoding genes with different bacterial species, within their hosts and in the environment [110]. STEC strains resistant to β-lactams, aminoglycosides, fluoroquinolones, phenicols, and tetracyclines, among others, have been isolated worldwide from livestock, beef, and humans [36,111,112,113]. Despite the low detection of AMR genes registered in this study, most identified genes are associated resistance to critically important or highly important veterinary and human antibiotics [114,115]; representing a critical issue. This low content of resistance genes could be the result of national legislation concerning antimicrobial use in livestock. For example, in Chile the use of any kind of antimicrobials as growth promoters has been banned since 2006 [116], and as prophylactics since 2019 [117], whereas in Uruguay antimicrobial use for the same purpose has been prohibited in cattle and sheep feed since 2011 [118]. Nevertheless, to date, the use of some antibacterial agents as growth promoters in livestock are still permitted in Argentina and Brazil.

## 5. Conclusions

We report the genomic characteristics of STEC strains circulating in the livestock-beef-human interface in South America, highlighting the successful expansion of O157:H7 (ST11) and non-O157 (ST16/CC29-O111:H8, ST21/CC29-O26:H11, ST223/CC155-O113:H21, ST58/CC155, ST677-O174:H21, ST443 -O178:H19, ST2388-O15:H27, ST679-O163:H19, ST78, ST106, ST101, and ST297) clones, most likely favored by common activities, such as multilateral trade and travel. Worryingly, some of these STEC clones have been isolated from severe human infections, including HUS, representing a risk for food safety and public health. The logistic regression model indicated that STEC isolates from cattle are more likely to harbor *stx1* than those isolated from beef, and that *stx2* seems to be a common feature of STEC strains isolated from cattle, beef products, and humans in South America. Additionally, STEC strains from South American countries other than Argentina and Chile present a higher probability of carrying both types of *stx*. It is important to establish that this study was performed using publicly available genome sequences, which could represent a bias due to underreporting of STEC isolates in this geographical region, highlighting the importance of establishing integrated surveillance programs, both national and regional, ensuring a One Health approach. In summary, these findings support the need for continuous monitoring and surveillance of STEC strains in South America, not only focusing on the detection of O157:H7 serotypes, but also on resistance profiles, virulome, and STs. In this regard, genomic surveillance can be used to rapidly identify and prevent the spread of critical clones “from farm to table”.

## Figures and Tables

**Table 1:** Logistic regression models for *stx1* and *stx1* + *stx2* association with geographical location and isolation source of the 130 STEC whole genome sequencing (WGS) analyzed.

***stx1***				**95% CI**	
Variable	Categories	Odds Ratio	*p*-Value	Lower	Upper
Intercept		0.238	0.059	0.054	1.057
Geographical location	Argentina	Reference			
	Chile	0.657	0.640	0.113	3.832
	Other	3.997	0.060	0.944	16.914
Isolation source	Beef	Reference			
	Cattle	3.800	0.017	1.265	11.419
	Human	1.069	0.935	0.214	5.347
***stx1 + stx2***				**95% CI**	
Variable	Categories	Odds Ratio	*p*-Value	Lower	Upper
Intercept		0.176	0.046	0.032	0.968
Geographical location	Argentina	Reference			
	Chile	0.688	0.714	0.094	5.061
	Others	8.541	0.037	1.142	63.859
Isolation source	Beef	Reference			
	Cattle	4.125	0.020	1.255	13.557
	Human	0.215	0.136	0.028	1.619

## Data Availability

Given that in this study only bacterial strains and STEC whole genome sequences were used, no ethical permissions were required. Whole-genome sequences of STEC strains sequenced here are deposited in GenBank under BioProject numbers PRJNA656305 and PRJNA682583.

## Supplementary Materials

The following are available online at https://www.mdpi.com/article/10.3390/ani11071845/s1. Table S1: Name, Sequence Read Archive (SRA) run accession numbers, collection information and ID species results for all 130 STEC strains isolated from cattle, beef and humans analyzed in this study. Table S2: Source, origin, isolation year, phylogroup, sequence type (ST), clonal complex (CC), serotype, virulome, AMR genes of 130 STEC strains isolated from cattle, beef and humans in South America deposited at GenBank. Table S3: SNP matrix STEC Chile.
