# Supplementary material for: Genomic Epidemiology of Shiga Toxin-Producing Escherichia coli Isolated from the Livestock-Food-Human Interface in South America
Source: Animals (Basel). 2021 Jun 22;11(7):1845. doi: 10.3390/ani11071845 (PMC8300192; doi:10.3390/ani11071845)
Supplement: Supplementary file 1 [file animals-11-01845-s001.zip › Supplementary material.pdf]

Table S1. Name, Sequence Read Archive (SRA) run accession numbers, collection information and ID species results for all 130 STEC strains isolated from cattle, beef and people analyzed in this study.

Strain	Run	Collected by	Species ID
MOD1-EC5234	SRR3993747	Penn State E. coli Reference Center	*Escherichia coli* / *Shigella*, 99.34%
ARIMEC38-12_C1	SRR6222011	Malbran	*Escherichia coli* / *Shigella*, 95.7%
ARIMEC110-16-1M	SRR6222063	Malbran	*Escherichia coli* / *Shigella*, 98.83%
ARIMEC14-88M	SRR6222230	Malbran	*Escherichia coli* / *Shigella*, 99.63%
ARIMEC160-17	SRR6222303	Malbran	*Escherichia coli* / *Shigella*, 100%
ARIMEC855-11	SRR6222306	Malbran	*Escherichia coli* / *Shigella*, 100%
ARIMEC680-04	SRR6222456	Malbran	*Escherichia coli* / *Shigella*, 99.28%
ARIMEC286-17	SRR6222461	Malbran	*Escherichia coli* / *Shigella*, 100%
ARIMEC707-08	SRR6222542	Malbran	*Escherichia coli* / *Shigella*, 97.44%
ARIMEC731-07	SRR6222558	Malbran	*Escherichia coli* / *Shigella*, 98.86%
ARIMEC171-98	SRR6236803	Malbran	*Escherichia coli* / *Shigella*, 99.65%
ARIMEC157-15-3M	SRR6236805	Malbran	*Escherichia coli* / *Shigella*, 99.64%
ARIMEC157-15-2M	SRR6236812	Malbran	*Escherichia coli* / *Shigella*, 99.62%
EC-0009	SRR6325149	Malbran	*Escherichia coli* / *Shigella*, 98.88%
ARIMEC60-11	SRR6425171	Malbran	*Escherichia coli* / *Shigella*, 100%
MOD1-EC5236	SRR6428552	Penn State E. coli Reference Center	*Escherichia coli* / *Shigella*, 99.09%
ARIM-EXH1186-06	SRR2015682	Malbran Institute-Argentina	*Escherichia coli* / *Shigella*, 99.53%
FWSEC0043	SRR7947248	Laboratory for Foodborne Zoonoses, Public Health Agency of Canada	*Escherichia coli* / *Shigella*, 98.8%
CFSAN027343	SRR8333591	E. coli Reference Center - Pennsylvania State University, University Park, PA	*Escherichia coli* / *Shigella*
ARIM-EXH737-14	SRR2015244	Malbran Institute-Argentina	*Escherichia coli* / *Shigella*, 100%

ARIM-EXH831-14.6	SRR2015665	Malbran Institute-Argentina	*Escherichia coli* / *Shigella*, 99.77%
ARIM-EXH831-14.5	SRR2015803	Malbran Institute-Argentina	*Escherichia coli* / *Shigella*, 99.87%
ARIM-EXH625-08	SRR2016400	Malbran Institute-Argentina	*Escherichia coli* / *Shigella*, 100%
ARIM-EXH485-02	SRR2015685	Malbran Institute-Argentina	*Escherichia coli* / *Shigella*, 100%
ARIM-EXH346-02	SRR2015792	Malbran Institute-Argentina	*Escherichia coli* / *Shigella*, 100%
ARIM-EXH368-07	SRR2015802	Malbran Institute-Argentina	*Escherichia coli* / *Shigella*, 99.85%
ARIM-EXH1133-06	SRR2015826	Malbran Institute-Argentina	*Escherichia coli* / *Shigella*, 100%
ARIM-EXH668-08	SRR2015683	Malbran Institute-Argentina	*Escherichia coli* / *Shigella*, 99.77%
ARIM-EXH644-08	SRR2015804	Malbran Institute-Argentina	*Escherichia coli* / *Shigella*, 100%
ARIM-EXH279-06	SRR2016393	Malbran Institute-Argentina	*Escherichia coli* / *Shigella*, 100%
CFSAN066347	SRR6764066	FDA	*Escherichia coli* / *Shigella*, 99.13%
CFSAN066346	SRR6764067	FDA	*Escherichia coli* / *Shigella*, 99.54%
CFSAN066388	SRR6764068	FDA	*Escherichia coli* / *Shigella*, 99.76%
CFSAN066334	SRR6764070	FDA	*Escherichia coli* / *Shigella*, 95.44%
CFSAN066341	SRR6764073	FDA	*Escherichia coli* / *Shigella*, 78.11%
CFSAN066340	SRR6764074	FDA	*Escherichia coli* / *Shigella*, 95.39%
CFSAN066345	SRR6764075	FDA	*Escherichia coli* / *Shigella*, 95.67%
CFSAN066342	SRR6764076	FDA	*Escherichia coli* / *Shigella*, 98.16%
CFSAN066314	SRR6764077	FDA	*Escherichia coli* / *Shigella*, 98.02%
CFSAN066313	SRR6764078	FDA	*Escherichia coli* / *Shigella*, 99.8%
CFSAN066312	SRR6764079	FDA	*Escherichia coli* / *Shigella*, 99.71%
CFSAN066311	SRR6764080	FDA	*Escherichia coli* / *Shigella*, 100%
CFSAN066319	SRR6764081	FDA	*Escherichia coli* / *Shigella*, 93.61%
CFSAN066317	SRR6764082	FDA	*Escherichia coli* / *Shigella*, 94.12%
CFSAN066316	SRR6764083	FDA	*Escherichia coli* / *Shigella*, 98.94%
CFSAN066315	SRR6764084	FDA	*Escherichia coli* / *Shigella*, 99.28%
CFSAN066372	SRR6764085	FDA	*Escherichia coli* / *Shigella*, 97.49%

CFSAN066371	SRR6764086	FDA	*Escherichia coli* / *Shigella*, 97.58%
CFSAN066322	SRR6764087	FDA	*Escherichia coli* / *Shigella*, 100%
CFSAN066320	SRR6764088	FDA	*Escherichia coli* / *Shigella*, 95.58%
CFSAN066368	SRR6764089	FDA	*Escherichia coli* / *Shigella*, 94.45%
CFSAN066367	SRR6764090	FDA	*Escherichia coli* / *Shigella*, 93.27%
CFSAN066366	SRR6764091	FDA	*Escherichia coli* / *Shigella*, 96.64%
CFSAN066365	SRR6764092	FDA	*Escherichia coli* / *Shigella*, 97.6%
CFSAN066370	SRR6764093	FDA	*Escherichia coli* / *Shigella*, 99.25%
CFSAN066309	SRR6764094	FDA	*Escherichia coli* / *Shigella*, 97.76%
CFSAN066302	SRR6764096	FDA	*Escherichia coli* / *Shigella*, 100%
CFSAN066301	SRR6764097	FDA	*Escherichia coli* / *Shigella*, 99.3%
CFSAN066303	SRR6764099	FDA	*Escherichia coli* / *Shigella*, 99.45%
CFSAN066306	SRR6764100	FDA	*Escherichia coli* / *Shigella*, 100%
CFSAN066308	SRR6764102	FDA	*Escherichia coli* / *Shigella*, 98.02%
CFSAN066307	SRR6764103	FDA	*Escherichia coli* / *Shigella*, 96.73%
CFSAN066390	SRR6764104	FDA	*Escherichia coli* / *Shigella*, 99.33%
CFSAN066391	SRR6764105	FDA	*Escherichia coli* / *Shigella*, 97.99%
CFSAN066386	SRR6764106	FDA	*Escherichia coli* / *Shigella*, 98.4%
CFSAN066387	SRR6764108	FDA	*Escherichia coli* / *Shigella*, 98.86%
CFSAN066332	SRR6764110	FDA	*Escherichia coli* / *Shigella*, 98.1%
CFSAN066326	SRR6764114	FDA	*Escherichia coli* / *Shigella*, 99.29%
CFSAN066323	SRR6764115	FDA	*Escherichia coli* / *Shigella*, 96.76%
CFSAN066324	SRR6764116	FDA	*Escherichia coli* / *Shigella*, 98%
CFSAN066329	SRR6764117	FDA	*Escherichia coli* / *Shigella*, 97.79%
CFSAN066327	SRR6764119	FDA	*Escherichia coli* / *Shigella*, 99.33%
CFSAN066328	SRR6764120	FDA	*Escherichia coli* / *Shigella*, 96.59%
CFSAN066360	SRR6764121	FDA	*Escherichia coli* / *Shigella*, 100%

CFSAN066380	SRR6764124	FDA	*Escherichia coli* / *Shigella*, 98.48%
CFSAN066378	SRR6764126	FDA	*Escherichia coli* / *Shigella*, 98.47%
CFSAN066375	SRR6764127	FDA	*Escherichia coli* / *Shigella*, 99.75%
CFSAN066349	SRR6764129	FDA	*Escherichia coli* / *Shigella*, 99.07%
CFSAN066354	SRR6764131	FDA	*Escherichia coli* / *Shigella*, 100%
CFSAN066355	SRR6764132	FDA	*Escherichia coli* / *Shigella*, 97.79%
CFSAN066357	SRR6764134	FDA	*Escherichia coli* / *Shigella*, 96.85%
CFSAN066359	SRR6764136	FDA	*Escherichia coli* / *Shigella*, 100%
CFSAN066381	SRR6764137	FDA	*Escherichia coli* / *Shigella*, 98.46%
CFSAN066382	SRR6764138	FDA	*Escherichia coli* / *Shigella*, 99.09%
CFSAN066399	SRR6764140	FDA	*Escherichia coli* / *Shigella*, 99.3%
CFSAN066389	SRR6764143	FDA	*Escherichia coli* / *Shigella*, 100%
CM 42-16	A-9	Dr. Nicolás Galarce	*Escherichia coli* / *Shigella*, 100%
CM 118-12	A-10	Dr. Nicolás Galarce	*Escherichia coli* / *Shigella*, 98.35%
CM 118-15	A-11	Dr. Nicolás Galarce	*Escherichia coli* / *Shigella*, 98.31%
CM 118-18	A-12	Dr. Nicolás Galarce	*Escherichia coli* / *Shigella*, 98.22%
CV 96-1	A-14	Dr. Nicolás Galarce	*Escherichia coli* / *Shigella*, 98.24%
CV 96-2	A-15	Dr. Nicolás Galarce	*Escherichia coli* / *Shigella*, 96.5%
CV 96-3	A-16	Dr. Nicolás Galarce	*Escherichia coli* / *Shigella*, 98.16%
CV 96-4	A-17	Dr. Nicolás Galarce	*Escherichia coli* / *Shigella*, 99.45%
CV 97-9	A-24	Dr. Nicolás Galarce	*Escherichia coli* / *Shigella*, 98.22%
CV 97-12	A-25	Dr. Nicolás Galarce	*Escherichia coli* / *Shigella*, 79.61%
CV 97-14	A-26	Dr. Nicolás Galarce	*Escherichia coli* / *Shigella*, 96.88%
B-6	B-6	Dr. Nicolás Galarce	*Escherichia coli* / *Shigella*, 100%
B-8	B-8	Dr. Nicolás Galarce	*Escherichia coli* / *Shigella*, 98.95%
B-12	B-12	Dr. Nicolás Galarce	*Escherichia coli* / *Shigella*, 99.53%
B-13	B-13	Dr. Nicolás Galarce	*Escherichia coli* / *Shigella*, 100%

B-14	B-14	Dr. Nicolás Galarce	*Escherichia coli* / *Shigella*, 98.89%
B-16	B-16	Dr. Nicolás Galarce	*Escherichia coli* / *Shigella*, 99.63%
B-17	B-17	Dr. Nicolás Galarce	*Escherichia coli* / *Shigella*, 100%
B-18	B-18	Dr. Nicolás Galarce	*Escherichia coli* / *Shigella*, 99.61%
B-19	B-19	Dr. Nicolás Galarce	*Escherichia coli* / *Shigella*, 99.68%
B-20	B-20	Dr. Nicolás Galarce	*Escherichia coli* / *Shigella*, 97.64%
B-22	B-22	Dr. Nicolás Galarce	*Escherichia coli* / *Shigella*, 99.34%
B-32	B-32	Dr. Nicolás Galarce	*Escherichia coli* / *Shigella*, 100%
B-40	B-40	Dr. Nicolás Galarce	*Escherichia coli* / *Shigella*, 100%
B-42	B-42	Dr. Nicolás Galarce	*Escherichia coli* / *Shigella*, 100%
B-44	B-44	Dr. Nicolás Galarce	*Escherichia coli* / *Shigella*, 100%
B-57	B-57	Dr. Nicolás Galarce	*Escherichia coli* / *Shigella*, 98.8%
B-58	B-58	Dr. Nicolás Galarce	*Escherichia coli* / *Shigella*, 100%
B-60	B-60	Dr. Nicolás Galarce	*Escherichia coli* / *Shigella*, 100%
B-61	B-61	Dr. Nicolás Galarce	*Escherichia coli* / *Shigella*, 96.97%
B-64	B-64	Dr. Nicolás Galarce	*Escherichia coli* / *Shigella*, 96.09%
RA-13	RA-13	Dr. Raúl Alegría	*Escherichia coli* / *Shigella*, 99.32%
R109_1	SRR7693849	Instituto de Microbiologia, Universidad San Francisco de Quito	*Escherichia coli* / *Shigella*, 100%
B196_1	SRR7693834	Instituto de Microbiologia, Universidad San Francisco de Quito	*Escherichia coli* / *Shigella*, 100%
EC-0015	SRR6325067	Malbran	*Escherichia coli* / *Shigella*, 99.66%
EC-0011	SRR6325070	Malbran	*Escherichia coli* / *Shigella*, 99.2%
EC-0016	SRR6325372	Malbran	*Escherichia coli* / *Shigella*, 100%
IHSV7	SRR12346095	Instituto de Higiene	*Escherichia coli* / *Shigella*, 98.3%
IHSV24	SRR12346096	Instituto de Higiene	*Escherichia coli* / *Shigella*, 99.13%

IHSV50	SRR12346097	Instituto de Higiene	*Escherichia coli* / *Shigella*, 99.23%
MOD1-EC1672	SRR5330931	Michigan State University	*Escherichia coli* / *Shigella*, 99.61%
IMP 871	SRR5927221	USDA ARS Meat Animal Research Center	*Escherichia coli* / *Shigella*, 100%
IMP 886	SRR5927222	USDA ARS Meat Animal Research Center	*Escherichia coli* / *Shigella*, 98.07%
IMP 1147	SRR6952375	USDA ARS Meat Animal Research Center	*Escherichia coli* / *Shigella*, 100%

Table S2. Source, origin, isolation year, phylogroup, sequence type (ST), clonal complex (CC), serotype, virulome, and AMR genes of 130 STEC strains isolated from cattle, beef and humans in South American countries (Genomes deposited at GenBank database).

Strain ID	Source	Country	Year	Phy[a]	ST (CC)[b]	Serotype	Virulome (LAA genes)[c]	AMR genes
MOD1-EC5234	Human	Argentina	1999	E	11 (11)	O157:H7	*stx2a, eae, ehaA, ehxA, hlyA, iha*	bla_{AmpC}
ARIMEC38-12_C1	Beef	Argentina	2012	B1	332	-O:H2	*stx2c, ehaA, hra, iha, lpfA, tia,* (*hes, lesP, pagC*-like)	bla_{AmpC}
ARIMEC110-16-1M	Human	Argentina	2013	E	11 (11)	O157:H7	*stx2a, eae, ehaA, ehxA, hlyA, iha*	bla_{AmpC}
ARIMEC14-88M	Human	Argentina	1988	E	11 (11)	-O:H7	*stx2a, eae, ehaA, ehxA, hlyA, iha*	bla_{AmpC}
ARIMEC160-17	Human	Argentina	2017	E	11 (11)	O157:H7	*stx2a, eae, ehaA, ehxA, hlyA, iha*	bla_{AmpC}
ARIMEC855-11	Beef	Argentina	2011	B1	6661 (469)	O8:H19	*stx1a, stx2a, ehaA, ehxA, hlyA, iha, lpfA*	bla_{AmpC}
ARIMEC680-04	Beef	Argentina	2004	E	11 (11)	O157:H7	*stx2a, eae, ehaA, ehxA, hlyA, iha*	bla_{AmpC}
ARIMEC286-17	Human	Argentina	2017	E	11 (11)	O157:H7	*stx2a, eae, ehaA, ehxA, hlyA, iha*	bla_{AmpC}
ARIMEC707-08	Human	Argentina	2008	B1	679 (469)	O163:H19	*stx2d, cdtB, ehaA, hra, iha, lpfA, tia,* (*ag43, hes, lesP, pagC*-like)	bla_{AmpC}
ARIMEC731-07	Beef	Argentina	2007	B1	677	O174:H21	*stx2c, ehaA, hra, iha, lpfA, tia*(*ag43, hes, lesP, pagC*-like)	bla_{AmpC}
ARIMEC171-98	Human	Argentina	1998	E	11 (11)	-O:H7	*stx1a, stx2a, eae, ehaA, ehxA, hlyA, iha*	bla_{AmpC}
ARIMEC157-15-3M	Human	Argentina	2015	E	11 (11)	-O:H7	*stx2a, eae, ehaA, iha*	bla_{AmpC}
ARIMEC157-15-2M	Human	Argentina	2015	E	11 (11)	O157:H7	*stx2a, eae, ehaA, ehxA, hlyA, iha*	bla_{AmpC}
EC-0009	Human	Argentina	2017	E	11 (11)	O157:H7	*stx2a, eae, ehaA, ehxA, hlyA, iha*	bla_{AmpC}
ARIMEC60-11	Human	Argentina	2011	E	11 (11)	O157:H7	*sx1a, eae, ehaA, iha*	bla_{AmpC}
MOD1-EC5236	Human	Argentina	1999	B1	1967 (20)	O103:H2	*stx1a, eae, efa1, ehaA, ehxA, hlyA*	bla_{AmpC}
ARIM-EXH1186-06	Human	Argentina	2006	E	11 (11)	-O:H7	*stx2a, eae, ehaA, iha*	bla_{AmpC}
FWSEC0043	Human	Argentina	1999	D	32 (32)	O145:H28	*stx2a, eae, ehaA, ehxA, hlyA, iha*	bla_{AmpC}

CFSAN027343	Human	Argentina	1999	B1	21 (29)	O26:H11	*stx1a, eae, efa1, ehaA, ehxA, hlyA, iha, lpfA*	*bla*$_{AmpC}$
ARIM-EXH737-14	Human	Argentina	2014	D	11 (11)	-O:H7	*stx2a, eae, ehaA, iha*	*bla*$_{AmpC}$
ARIM-EXH831-14.6	Beef	Argentina	2014	D	11 (11)	-O:H7	*stx2a, eae, ehaA, ehxA, iha*	*bla*$_{AmpC}$
ARIM-EXH831-14.5	Beef	Argentina	2014	D	11 (11)	-O:H7	*stx2a, eae, ehaA, iha*	*bla*$_{AmpC}$
ARIM-EXH625-08	Human	Argentina	2008	E	11 (11)	-O:H7	*stx1a, eae, ehaA, ehxA, hlyA, iha*	*bla*$_{AmpC}$
ARIM-EXH485-02	Beef	Argentina	2002	E	11 (11)	-O:H7	*stx2a, eae, ehaA, iha*	*bla*$_{AmpC}$
ARIM-EXH346-02	Human	Argentina	2002	E	11 (11)	-O:H7	*stx2a, eae, ehaA, iha*	*bla*$_{AmpC}$
ARIM-EXH368-07	Human	Argentina	2007	E	11 (11)	-O:H7	*stx2a, eae, ehaA, iha*	*bla*$_{AmpC}$
ARIM-EXH1133-06	Human	Argentina	2006	E	11 (11)	O157:H7	*stx2a, eae, ehaA, iha*	*bla*$_{AmpC}$
ARIM-EXH668-08	Beef	Argentina	2008	E	11 (11)	-O:H7	*stx2a, eae, ehaA, iha*	*bla*$_{AmpC}$
ARIM-EXH644-08	Human	Argentina	2008	E	11 (11)	-O:H7	*stx2a, eae, ehaA, iha*	*bla*$_{AmpC}$
ARIM-EXH279-06	Human	Argentina	2005	E	11 (11)	-O:H7	*stx2a, eae, ehaA, iha*	*bla*$_{AmpC}$
CFSAN066347	Cattle	Chile	2016	A	8134	O172:H25	*stx2a, eae, efa1, ehaA, lpfA*	*bla*$_{AmpC}$
CFSAN066346	Cattle	Chile	2016	A	8134	O172:H25	*stx2a, eae, efa1, ehaA, ehxA, hlyA, lpfA*	*bla*$_{AmpC}$
CFSAN066388	Cattle	Chile	2016	B1	21 (29)	O26:H11	*stx1a, eae, efa1, ehaA, ehxA, hlyA, lpfA*	*bla*$_{AmpC}$
CFSAN066334	Beef	Chile	2016	B1	8141 (156)	O174:H28	*stx2c, ehxA, hlyA, iha, lpfA, saa, sab, subA*, (*ag43, pagC*-like)	*bla*$_{AmpC}$
CFSAN066341	Cattle	Chile	2016	B1	223 (155)	O113:H21	*stx1a, stx2a, cdtB, ehaA, ehxA, hlyA, iha, lpfA, sab, subA*, (*ag43, pagC*-like)	*bla*$_{AmpC}$
CFSAN066340	Cattle	Chile	2016	B1	58 (155)	O116:H21	*stx2a, cdtB, ehaA, ehxA, hlyA, hra, iha, lpfA, saa, subA*, (*hes, pagC*-like)	*bla*$_{AmpC}$
CFSAN066345	Cattle	Chile	2016	B1	306	O98:H21	*stx1a, eae, ehaA, ehxA, hlyA, iha, lpfA*	*bla*$_{AmpC}$
CFSAN066342	Cattle	Chile	2016	B1	718 (446)	O168:H8	*stx2g, ehaA, ehxA, hlyA, lpfA*	*bla*$_{AmpC}$
CFSAN066314	Beef	Chile	2016	B1	297	O93:H46	*stx2c, ehaA, ehxA, hlyA, iha, lpfA, saa, subA*	*bla*$_{AmpC}$
CFSAN066313	Beef	Chile	2016	B1	2387	O185:H7	*stx2c, ehaA, hra, iha, lpfA, tia*, (*ag43, hes, pagC*-like)	*bla*$_{AmpC}$
CFSAN066312	Beef	Chile	2016	B1	297	O93:H46	*stx2c, ehaA, ehxA, iha, lpfA, saa, subA*	*bla*$_{AmpC}$

CFSAN066311	Beef	Chile	2016	B1	297	O93:H46	*stx2c, ehaA, ehxA, hlyA, iha, lpfA, saa, subA*	*bla*$_{AmpC}$
CFSAN066319	Beef	Chile	2016	B1	101 (101)	O82:H8	*stx1a, ehaA, ehxA, hlyA, iha, lpfA, saa, sab,* (*ag43, pagC*-like)	*bla*$_{AmpC}$
CFSAN066317	Beef	Chile	2016	B1	2458	O91:H21	*stx2a, cdtB, ehaA, ehxA, hlyA, hra, iha, lpfA, saa, tia,* (*ag43, hes, pagC*-like)	*bla*$_{AmpC}$
CFSAN066316	Beef	Chile	2016	B1	297	O93:H46	*stx2c, ehaA, ehxA, hlyA, iha, lpfA, saa, subA*	*bla*$_{AmpC}$
CFSAN066315	Beef	Chile	2016	B1	297	O93:H46	*stx2c, ehaA, ehxA, hlyA, iha, lpfA, saa, subA*	*bla*$_{AmpC}$
CFSAN066372	Cattle	Chile	2016	B1	58 (155)	O116:H21	*stx2a, ehaA, ehxA, hlyA, hra, iha, lpfA, saa, subA, tia,* (*hes, pagC*-like)	*bla*$_{AmpC}$
CFSAN066371	Cattle	Chile	2016	B1	58 (155)	O116:H21	*stx2a, ehaA, ehxA, hlyA, hra, iha, lpfA, saa, subA, tia,* (*hes, pagC*-like)	*bla*$_{AmpC}$
CFSAN066322	Beef	Chile	2016	B1	8138	O185:H7	*stx2c, ehaA, hra, iha, lpfA, tia,* (*ag43, hes, pagC*-like)	*bla*$_{AmpC}$
CFSAN066320	Beef	Chile	2016	B1	101 (101)	O82:H8	*stx1a, stx2a, ehaA, ehxA, hlyA, iha, lpfA, saa, sab,* (*ag43, pagC*-like)	*bla*$_{AmpC}$
CFSAN066368	Cattle	Chile	2016	B1	2458	O91:H21	*stx2a, cdtB, ehaA, ehxA, hlyA, hra, iha, lpfA, saa, tia*(*ag43, hes, pagC*-like)	*bla*$_{AmpC}$
CFSAN066367	Cattle	Chile	2016	B1	2458	O91:H21	*stx2a, cdtB, ehaA, ehxA, hlyA, hra, iha, lpfA, saa, tia,* (*ag43, hes, pagC*-like)	*bla*$_{AmpC}$
CFSAN066366	Cattle	Chile	2016	G	657	O183:H18	*stx1a, stx2a, cdtB, ehaA, ehxA, hlyA, iha, lpfA, saa*	*bla*$_{AmpC}$
CFSAN066365	Cattle	Chile	2016	B1	192	O178:H19	*stx2c, ehaA, hra, iha, lpfA, tia,* (*hes, pagC*-like)	*bla*$_{AmpC}$
CFSAN066370	Cattle	Chile	2016	B1	443 (205)	O178:H19	*stx1a, stx2a, ehaA, ehxA, hlyA, hra, iha, lpfA, saa, tia,* (*hes*)	*bla*$_{AmpC}$

CFSAN066309	Beef	Chile	2016	B1	677	O174:H21	*stx2c, ehaA, hra, iha, lpfA, tia*, (*ag43, hes, lesP, pagC*-like)	*aph(3'')-Ib, aph(6)-Id, bla*$_{AmpC}$, *tetB*
CFSAN066302	Beef	Chile	2016	B1	1125	-O:H19	*stx2a, ehaA, ehxA, hlyA, hra, iha, lpfA, saa, sab, subA, tia*, (*ag43, hes*)	*bla*$_{AmpC}$
CFSAN066301	Beef	Chile	2016	B1	1613	-O:H21	*stx1a, stx2c, ehaA, ehxA, hlyA, hra, iha, lpfA, saa, subA, tia*, (*ag43, hes, pagC*-like)	*bla*$_{AmpC}$
CFSAN066303	Beef	Chile	2016	B1	2387	O185:H7	*stx2c, ehaA, hra, iha, lpfA, tia*, (*ag43, hes, pagC*-like)	*bla*$_{AmpC}$
CFSAN066306	Beef	Chile	2016	B1	446 (446)	O22:H8	*stx2d, ehaA, iha, lpfA*	*bla*$_{AmpC}$
CFSAN066308	Beef	Chile	2016	B1	297	O93:H46	*stx2c, ehaA, ehxA, hlyA, iha, lpfA, saa, subA*	*bla*$_{AmpC}$
CFSAN066307	Beef	Chile	2016	B1	677	O174:H21	*stx2c, ehaA, hra, iha, lpfA, tia*, (*hes, lesP, pagC*-like)	*aph(3'')-Ib, aph(6)-Id, bla*$_{AmpC}$, *tetB*
CFSAN066390	Cattle	Chile	2016	B1	297	O130:H11	*stx2a, ehaA, ehxA, hlyA, iha, lpfA, saa, sab, subA*	*bla*$_{AmpC}$
CFSAN066391	Cattle	Chile	2016	B1	58 (155)	O116:H21	*stx2a, ehaA, ehxA, hlyA, hra, iha, lpfA, saa, subA, tia*, (*hes, pagC*-like)	*bla*$_{AmpC}$
CFSAN066386	Cattle	Chile	2016	B1	443 (205)	O178:H19	*stx1a, stx2a, ehaA, hra, iha, lpfA, saa, tia*, (*ag43, hes, lesP*)	*bla*$_{AmpC}$
CFSAN066387	Cattle	Chile	2016	B1	443 (205)	O178:H19	*stx1a, stx2a, ehaA, ehxA, hlyA, hra, iha, lpfA, saa, tia*, (*hes, lesP*)	*bla*$_{AmpC}$
CFSAN066332	Beef	Chile	2016	B1	223 (55)	O113:H21	*stx2a, cdtB, ehaA, ehxA, hlyA, hra, iha, lpfA, saa, sab, subA, tia*, (*ag43, hes, pagC*-like)	*bla*$_{AmpC}$
CFSAN066326	Beef	Chile	2016	B1	2388 (20)	O15:H27	*stx2d, ehaA, hra, iha, lpfA, tia*	*bla*$_{AmpC}$
CFSAN066323	Beef	Chile	2016	B1	332	O171:H2	*stx2c, ehaA, hra, iha, lpfA, tia*, (*hes, lesP, pagC*-like)	*bla*$_{AmpC}$
CFSAN066324	Beef	Chile	2016	B1	297	O93:H46	*stx2c, ehaA, ehxA, hlyA, iha, lpfA, sab, subA*	*bla*$_{AmpC}$
CFSAN066329	Beef	Chile	2016	B1	442	O91:H21	*stx2a, cdtB, ehaA, ehxA, hlyA, hra, iha, lpfA, tia*, (*ag43, hes, pagC*-like)	*bla*$_{AmpC}$

CFSAN066327	Beef	Chile	2016	B1	677	O174:H21	*stx2c, ehaA, hra, iha, lpfA, tia,* (*hes, lesP, pagC*-like)	*aph(3'')-Ib*, *aph(6)-Id*, *bla*AmpC, *sul*2, *tetB*
CFSAN066328	Beef	Chile	2016	B1	8135	O116:H21	*stx1a, stx2c, stx2d, cdtB, ehaA, ehxA, hlyA, hra, iha, lpfA, subA, tia,* (*ag43, hes, pagC*-like)	*bla*AmpC
CFSAN066360	Cattle	Chile	2016	B1	446 (446)	O22:H8	*stx2d, ehaA, iha, lpfA*	*bla*AmpC
CFSAN066380	Cattle	Chile	2016	B1	223 (155)	O113:H21	*stx2a, ehaA, ehxA, hlyA, hra, iha, lpfA, sab, subA, tia,* (*ag43, hes, pagC*-like)	*bla*AmpC
CFSAN066378	Cattle	Chile	2016	B1	332	O171:H2	*stx2c, ehaA, iha, lpfA,* (*ag43, lesP, pagC*-like)	*bla*AmpC
CFSAN066375	Cattle	Chile	2016	B1	443 (205)	O178:H19	*stx2c, ehaA, ehxA, hlyA, hra, iha, lpfA, saa, sab, tia,* (*ag43, hes, lesP*)	*bla*AmpC
CFSAN066349	Cattle	Chile	2016	A	8134	O172:H25	*stx2a, eae, efa1, ehaA, ehxA, hlyA, lpfA*	*bla*AmpC
CFSAN066354	Cattle	Chile	2016	B1	173	O181:H49	*stx2c, stx2d, ehxA, hlyA, iha, lpfA, saa, subA,* (*ag43, pagC*-like)	*bla*AmpC
CFSAN066355	Cattle	Chile	2016	B1	718 (446)	O168:H8	*stx2g, ehaA, ehxA, hlyA, lpfA*	*bla*AmpC
CFSAN066357	Cattle	Chile	2016	B1	332	O171:H2	*stx2a, ehaA, hra, iha, lpfA, tia,* (*hes, lesP, pagC*-like)	*bla*AmpC
CFSAN066359	Cattle	Chile	2016	B1	446 (446)	O22:H8	*stx2d, ehaA, iha, lpfA*	*bla*AmpC
CFSAN066381	Cattle	Chile	2016	B1	718 (446)	O168:H8	*stx2d, ehaA, hra, iha, lpfA, tia,* (*ag43, hes, lesP, pagC*-like)	*bla*AmpC
CFSAN066382	Cattle	Chile	2016	B1	223 (155)	O113:H21	*stx1a, stx2a, ehaA, ehxA, hlyA, iha, lpfA, sab, subA*	*bla*AmpC
CFSAN066399	Beef	Chile	2016	B1	56 (155)	O113:H21	*stx2b, stx2c, ehaA, hra, iha, lpfA, tia,* (*hes, pagC*-like)	*bla*AmpC
CFSAN066389	Cattle	Chile	2016	B1	297	O130:H11	*stx2a, ehaA, lpfA, subA*	*bla*AmpC
CM 42-16	Beef	Chile	2019	B1	54	O104:H2	*stx1a, stx2d, ehaA, ehxA, hlyA, iha, lpfA, saa,* (*ag43, pagC*-like)	*bla*AmpC
CM 118-12	Beef	Chile	2019	B1	58 (155)	O116:H21	*stx2a, cdtB, ehaA, hra, iha, lpfA, saa, subA, tia,* (*hes, pagC*-like)	*bla*AmpC, *qnrB19*

CM 118-15	Beef	Chile	2019	B1	58 (155)	O116:H21	*stx2a, cdtB, ehaA, hlyA, hra, iha, lpfA, saa, subA, tia*, (*hes, pagC*-like)	*bla*AmpC, *qnrB19*
CM 118-18	Beef	Chile	2019	B1	58 (155)	O116:H21	*stx2a, cdtB, ehaA, ehxA, hlyA, hra, iha, lpfA, saa, subA, tia*, (*hes, pagC*-like)	*bla*AmpC
CV 96-1	Beef	Chile	2019	B1	11231 (155)	O113:H21	*stx2a, cdtB, ehaA, ehxA, hlyA, hra, iha, lpfA, saa, sab, subA, tia*, (*ag43, hes, pagC*-like)	*bla*AmpC
CV 96-2	Beef	Chile	2019	B1	11231 (155)	O113:H21	*stx2a, cdtB, ehaA, ehxA, hlyA, hra, iha, lpfA, saa, sab, subA, tia*, (*ag43, hes, pagC*-like)	*bla*AmpC
CV 96-3	Beef	Chile	2019	B1	11231 (155)	O113:H21	*stx2a, cdtB, ehaA, ehxA, hlyA, hra, iha, lpfA, saa, sab, subA, tia*, (*ag43, hes, pagC*-like)	*bla*AmpC
CV 96-4	Beef	Chile	2019	B1	11231 (155)	O113:H21	*stx2a, cdtB, ehxA, hlyA, hra, iha, lpfA, saa, sab, subA, tia*, (*ag43, hes, pagC*-like)	*bla*AmpC
CV 97-9	Beef	Chile	2019	B1	11231 (155)	O113:H21	*stx2a, cdtB, ehaA, ehxA, hlyA, hra, iha, lpfA, saa, sab, subA, tia*, (*ag43, hes, pagC*-like)	*bla*AmpC
CV 97-12	Beef	Chile	2019	B1	-1635 (155)	O113:H21	*stx2a, cdtB, ehaA, ehxA, hlyA, hra, iha, lpfA, saa, sab, subA, tia*, (*ag43, hes, pagC*-like)	*bla*AmpC
CV 97-14	Beef	Chile	2019	B1	11231 (155)	O113:H21	*stx2a, cdtB, ehaA, ehxA, hlyA, hra, iha, lpfA, saa, sab, subA, tia*, (*ag43, hes, pagC*-like)	*bla*AmpC
B-6	Cattle	Chile	2018	B1	1125	-O:H19	*stx2a, ehaA, ehxA, hlyA, iha, lpfA, saa, sab, subA*	*bla*AmpC
B-8	Cattle	Chile	2018	B1	332	O171:H2	*stx2b, ehaA, hra, iha, lpfA, tia*, (*hes, lesP, pagC*-like)	*bla*AmpC
B-12	Cattle	Chile	2018	B1	692	O74:H42	*stx1a, stx2d, ehaA, hra, iha, lpfA, tia*, (*ag43, hes, lesP*)	*bla*AmpC
B-13	Cattle	Chile	2018	B1	33	O91:H14	*stx1a, stx2d, cdtB, ehaA, iha, lpfA, saa, subA, tia*	*bla*AmpC

B-14	Cattle	Chile	2018	B1	33	O91:H14	*stx1a, stx2d, cdtB, ehaA, iha, lpfA, saa, subA, tia*	*bla*$_{AmpC}$
B-16	Cattle	Chile	2018	B1	-1661	O91:H14	*stx1a, stx2d, cdtB, ehaA, iha, lpfA, saa, subA, tia*, (*ag43*)	*bla*$_{AmpC}$
B-17	Cattle	Chile	2018	B1	332	O171:H2	*stx2c, stx2d, ehaA, hra, iha, lpfA, tia*, (*hes, lesP, pagC*-like)	*bla*$_{AmpC}$
B-18	Cattle	Chile	2018	B1	448 (448)	O148:H8	*stx2c, ehaA, iha, lpfA, tia*	*bla*$_{AmpC}$
B-19	Cattle	Chile	2018	B1	448 (448)	O148:H8	*stx2c, iha, lpfA, tia*	*bla*$_{AmpC}$
B-20	Cattle	Chile	2018	B1	448 (448)	O148:H8	*stx2c, ehaA, iha, lpfA, tia*	*bla*$_{AmpC}$
B-22	Cattle	Chile	2018	B1	1248	-O:H21	*stx1a, stx2d, ehaA, ehxA, hlyA, iha, lpfA, saa*, (*ag43, pagC*-like)	*bla*$_{AmpC}$
B-32	Cattle	Chile	2018	B1	297	O130:H11	*stx1a, stx2d, ehaA, ehxA, hlyA, iha, lpfA, saa, subA*	*bla*$_{AmpC}$
B-40	Cattle	Chile	2018	B1	297	O130:H11	*stx2a, ehaA, ehxA, hlyA, iha, lpfA, saa, sab, subA*	*bla*$_{AmpC}$
B-42	Cattle	Chile	2018	B1	446 (446)	O22:H8	*stx2d, ehaA, iha, lpfA*	*bla*$_{AmpC}$
B-44	Cattle	Chile	2018	B1	443 (205)	O178:H19	*stx1a, stx2a, ehaA, ehxA, hlyA, hra, iha, lpfA, saa, tia*, (*hes*)	*bla*$_{AmpC}$
B-57	Cattle	Chile	2018	G	657	O183:H18	*stx1a, stx2a, ehaA, ehxA, hlyA, iha, lpfA, saa*	*bla*$_{AmpC}$
B-58	Cattle	Chile	2018	B1	1125	-O:H19	*stx2d, cdtB, ehaA, ehxA, hlyA, iha, lpfA, saa, sab, subA*	*bla*$_{AmpC}$
B-60	Cattle	Chile	2018	B1	1125	-O:H19	*stx2d, cdtB, ehaA, ehxA, hlyA, iha, lpfA, saa, sab, subA*	*bla*$_{AmpC}$
B-61	Cattle	Chile	2018	G	11238	O183:H18	*stx1a, stx2a, ehaA, ehxA, hlyA, iha, lpfA, saa*	*bla*$_{AmpC}$
B-64	Cattle	Chile	2018	G	657	O183:H18	*stx1a, stx2a, ehaA, ehxA, hlyA, iha, lpfA, saa*	*bla*$_{AmpC}$
RA-13	Cattle	Chile	2019	B1	297	O130:H11	*stx1a, stx2d, ehaA, iha, lpfA, saa, sab, subA*	*bla*$_{AmpC}$
R109_1	Human	Ecuador	2015	B1	16 (29)	O111:H8	*stx1a, eae, ehaA, iha, lpfA*	*bla*$_{AmpC}$
B196_1	Human	Ecuador	2015	B1	21 (29)	O69:H11	*stx1a, eae, efa1, ehaA, ehxA, hlyA, lpfA*	*bla*$_{AmpC}$
EC-0015	Human	Paraguay	2009	E	11 (11)	O157:H7	*stx2a, eae, ehaA, iha*	*bla*$_{AmpC}$, *bla*$_{TEM-1B}$
EC-0011	Human	Paraguay	2003	E	11 (11)	O157:H7	*stx2c, eae, ehaA, iha*	*bla*$_{AmpC}$

EC-0016	Human	Paraguay	2009	E	11 (11)	O157:H7	*stx2a, eae, ehaA, iha*	bla_{AmpC}, $bla_{\text{TEM-1B}}$
IHSV7	Human	Uruguay	2017	B1	16 (29)	O111:H8	*stx1a, stx2a, eae, efa1, ehaA, ehxA, hlyA, iha, lpfA*	*aph(3′)-Ia, aph(3′′)-Ib*, bla_{AmpC}, $bla_{\text{TEM-1B}}$, *sul2, tetA*
IHSV24	Human	Uruguay	2011	E	11 (11)	O157:H7	*stx1a, stx2a, eae, ehaA, iha*	bla_{AmpC}
IHSV50	Human	Uruguay	2016	A	342	O145:H25	*stx2a, eae, efa1, ehxA, hlyA, lpfA*	bla_{AmpC}, *fosA7*
MOD1-EC1672	Human	Uruguay	-	A	342	O145:H25	*stx2a, eae, efa1, ehaA, ehxA, hlyA, lpfA*	bla_{AmpC}, *fos*A7
IMP 871	Beef	Uruguay	2004	B1	679 (469)	O163:H19	*stx2a, cdtB, ehaA, ehxA, hlyA, hra, iha, lpfA, saa, subA, tia*, (*ag43, hes, pag*C-like)	bla_{AmpC}
IMP 886	Beef	Uruguay	2005	B1	443 (205)	O178:H19	*stx1a, stx2a, ehaA, ehxA, hlyA, hra, iha, lpfA, saa, tia*, (*hes*)	bla_{AmpC}
IMP 1147	Beef	Uruguay	2005	B1	2388 (20)	O15:H27	*stx1c, stx2d, ehaA, hra, iha, lpfA, tia*	bla_{AmpC}

[a] Phylogenetic group determined *in silico* by Clermont typing (http://clermontyping.iame-research.center/)

[b] Strain B-16 belongs to a novel ST [single locus variant (SLV) of ST1661 at the *fumC* allele].

[c] Virulence genes associated with Locus of Adhesion and Autoaggregation (LAA).
